# Synthetic Preimplantation Factor (sPIF)* Improves Survival and GI Function in Acute Lethal Radiation Syndrome in a Clinically Relevant Murine Model

**DOI:** 10.3390/ijms27177548

**Published:** 2026-08-23

**Authors:** Roberto Calix, Jean H. Wilson, Martin Mueller, Peter M. Glazer, Daniel Zelterman, Michelle Deveaux, Caroline Zeiss, Arumugam R. Jayakumar, Eytan R. Barnea, Michael J. Paidas

**Affiliations:** 1 Section of Maternal Fetal Medicine, Department of Obstetrics, Gynecology & Reproductive Sciences, Yale School of Medicine, 333 Cedar Street, FMB 329, P.O. Box 208063, New Haven, CT 06520-8063, USA; roberto.calix@yale.edu; 2 Maternal and Fetal Medicine, Ochsner Health, 2700 Napoleon Ave. 4th Floor, New Orleans, LA 70115, USA; 3 Yale Women and Children’s Center for Blood Disorders and Preeclampsia Advancement, Department of Obstetrics, Gynecology and Reproductive Sciences, Yale School of Medicine, 310 Cedar Street, FMB 220, New Haven, CT 06520-8063, USA; jean.wilson@yale.edu; 4 Department of Obstetrics, Gynecology and Reproductive Sciences, Yale School of Medicine, 333 Cedar Street, FMB 337A, New Haven, CT 06520-8063, USA; martin.mueller@unibe.ch; 5 Institute of Biochemistry and Molecular Medicine, University of Bern, 3010 Bern, Switzerland; 6 Department of Therapeutic Radiology, Yale School of Medicine, P.O. Box 208040, New Haven, CT 06520-8040, USA; peter.glazer@yale.edu; 7 Yale School of Public Health, 60 College Street, P.O. Box 208034, New Haven, CT 06510-3201, USA; daniel.zelterman@yale.edu (D.Z.); michelle.deveaux@yale.edu (M.D.); 8 Department of Early Clinical Development, Regeneron Pharmaceuticals, Tarrytown, NY 10591-6717, USA; 9 Department of Comparative Medicine, Yale School of Medicine, P.O. Box 208016, New Haven, CT 06520-8016, USA; caroline.zeiss@yale.edu; 10 Tamer Family Department of Obstetrics, Gynecology, and Reproductive Sciences, Miller School of Medicine, University of Miami, 1120 NW 14th Street, Suite # 1154, Miami, FL 33136-2107, USA; ajayakumar@med.miami.edu; 11 BioIncept, LLC (*sPIF Proprietary), New York, NY 10016-1701, USA

**Keywords:** Synthetic Preimplantation Factor (sPIF*), hematopoietic and gastrointestinal ARS, radiation injury, therapy, *Proprietary, BioIncept, LLC, New York

## Abstract

Ionizing radiation (IR) damage, whether intentional, accidental, or therapeutic, reverberates throughout the body. The FDA has approved the use of palliative care and leukocyte growth factors for hematologic depletion (H-ARS), but no treatments have been approved for gastrointestinal damage (GI-ARS). Synthetic PIF (sPIF) safely replicates endogenous PIF’s preventative, reparatory, and regenerative effects. Specifically relevant for radiation-induced damage, sPIF reduces oxidative stress. Subcutaneous (SC) sPIF prevents LD100/30 mortality at 2 w post-treatment and restores GI function post-6Gy exposure (in mice). Daily SC sPIF for 14 d, starting 24 h post-LD85/40 (7.17 Gy) total-body ɣ-irradiation (TBI), increases survival 2.76-fold (14.7% (5/34) to 40.7% (24/59); *p* = 0.0036) by 4 w post-therapy. All sPIF-treated mice survived for the first 10 d, while only 80% survived in the sham group (*p* = 0.0034). The sPIF-treated group reached 50% survival at 23 di, while this figure was reached at 19 d in the sham-treated group, with a 21% delay. Importantly, sPIF delays weight loss for up to 25 d, increasing weight by ~12% above baseline and improving colon architecture; in the sham, both weight and colon recovery failed. sPIF partially prevents declines in hemoglobin and platelets, with no bleeding/sepsis observed, while WBC counts declined. sPIF’s safety and lack of deleterious drug-to-drug interaction were documented in a First-in-Human, FDA-approved clinical trial for autoimmune disease. Chronic FDA-guided toxicology/toxicokinetic studies demonstrated NOAEL and the highest safety margin. Collectively, sPIF safely and significantly increases survival, weight gain, and colon crypt scale, directly supporting its suitability for FDA-ARS animal-rule approval.

## 1. Introduction

Acute radiation syndrome (ARS) constitutes a spectrum of clinical signs and symptoms involving the hematopoietic (H), gastrointestinal (GI), neurovascular, and/or cutaneous and vascular damage that develop after exposure to ionizing radiation (IR) [1,2,3]. The severity of signs and symptoms correlates with the absorbed total-body irradiation (TBI) dose [4,5]. A classification system for radiation injury was developed based on the degree of toxicity to each of the four organ systems above [6]. Without intervention, IR-induced damage starts at >1 Gy; at doses exceeding 5–6 Gy, the observed high mortality is due to infection, bleeding, and multi-organ dysfunction and/or failure [7]. Surviving IR due to radiological accidents, weapons of mass destruction, or therapeutic exposure is dependent on the duration of exposure, the amount of time that has passed before the first treatment, the dose received, and the distance from the radiation source [8]. Whereas local radiotherapy is a commonly used treatment for cancer, current world events highlight the urgent need to develop new, safe comprehensive agents that (i) mitigate the deleterious effects of IR, especially TBI; (ii) prolong survival in ARS; (iii) exhibit stability for stockpiling and use in or near the radiation field or in local medical treatment facilities; and (iv) are stable at ambient temperature and easy to administer [9,10].

The underlying pathophysiology for radiation injury from exposure > 1 Gy includes damage to stem cells located in the bone marrow, circulation, skin, and crypts of the gastrointestinal tract [1,2,3,6,11]. Injuries affecting hematopoietic stem cells result in H-ARS, a clinical diagnosis characterized by a reduction in white blood cell populations (with lymphocytes being the most susceptible), platelets, and red blood cells [12,13,14]. Neutropenia and thrombocytopenia may be life-threatening, leading to death by infection and/or bleeding. Administration of antibiotics and leukocyte growth factors (LGFs) and transfusion of blood products may improve morbidity and delay IR-induced death [12]. The onset of lethal GI—and cerebrovascular—ARS is associated with a higher dose of radiation than that for H-ARS, but symptoms of both can occur at lower doses as well [10,11].

Current treatments for H-ARS include the LGFs Neupogen^®^, Neulasta^®^, and Leukine^®^, as well as related analogs, which are the only US Food and Drug Administration (FDA)-approved drugs for radiation-induced myelosuppression resulting from exposure during a radiological or nuclear incident [3,15,16]. These LGFs promote myeloid stem cell/progenitor cell differentiation and proliferation, leading to the re-population of neutrophils in the circulation and peripheral tissues [17]. In chemotherapy-treated patients with cancer, LGFs reduce the risk of infection and sepsis [2,3,6]. Although hematopoietic stem cell transplantation (HSCT) is theoretically efficacious after stem cell ablation following IR, the results of this approach in accidentally irradiated individuals have thus far been suboptimal [3]. Frequent complications, including graft-versus-host disease (GvHD), despite attempts to match the transplanted bone marrow, contribute to this limited utility [18]. Nevertheless, some individuals who fail to respond to LGFs may be candidates for HSCT.

Management of GI-ARS remains an unmet need because there are no approved drugs for treating this syndrome [19]. GI-ARS symptomatology begins with an early loss of epithelial crypts and progresses to ulceration. Later, diarrhea, constipation, fibrosis, and fistula formation occur. Inevitably, such GI pathologies lead to malnutrition, a requirement for parenteral nutrition and hydration, and associated weight loss that can become permanent [20]. Current approaches to identifying MCM drugs against GI-ARS involve the use of TBI animal models, with or without partial bone marrow shielding [10].

It is widely recognized that healthcare providers urgently need to develop new mitigators of ARS [3]. However, potential ARS therapies for individuals exposed to TBI during a mass-casualty incident are difficult to study in humans because appropriate controls are lacking [21]. In addition, among patients undergoing radiotherapy, exposure (e.g., targeted regional radiotherapy) is supposed to be minimal, so they are not suitable as control populations [2]. A confounding factor is the spontaneous recovery observed in some patients without cytokine or stem cell therapy, even after the development of grade 4 H-ARS [22].

To address these issues, the FDA developed the “Animal Rule,” which requires that a drug be approved to protect against ARS when human efficacy and field-trial studies are not practical or ethical. This requires 1. prevention or a significant reduction in ARS pathophysiology; 2. two animal species, unless one is clearly effective; 3. a clinical endpoint of prevention or increased survival; and 4. established kinetics and pharmacodynamics in animals and humans [23]. Ideally, new treatments for ARS must be safe, effective, easy to administer, stable at ambient and extreme temperatures, and readily available in sufficient quantities for treating many individuals who have potentially been exposed to a myelosuppressive dose of IR.

In this context, synthetic preimplantation factor (sPIF) may possess the features required to become a safe and effective therapeutic agent [24]. Endogenous PIF is an evolutionarily conserved, 15-amino-acid peptide only secreted by viable mammalian embryos [25,26,27]. Through autotrophic action, the peptide promotes embryo development and protects against oxidative stress and cellular damage [28,29]. PIF interacts with the maternal host to promote implantation (through trophoblast invasion via p53 anti-apoptotic pathways) and protects against fetal death following bacterial antigen exposure [26,30,31,32,33,34,35]. The synthetic PIF (sPIF) molecule replicates endogenous immune-protective, preventative, reparative, and regenerative functions and exerts potent, integrated regulatory effects on both systemic immunity and local inflammation in the affected organ [36,37,38]. By binding to innate CD14+ cells (macrophages and neutrophils) and, following activation, the adaptive arm of the immune system, sPIF reduces proliferation in the mixed lymphocyte reaction (a basis for tolerance) and leads to a Th2/Th1 cytokine bias while preserving the critical antipathogenic effect by increasing the secretion of certain Th1 cytokines [36,37].

Mechanistically, sPIF is effective in nonpregnant immune disease models because it targets protein disulfide isomerase/thioredoxin (PDI/T) and heat shock proteins (HSPs), thereby reducing oxidative stress and protein misfolding and preventing cellular damage [38,39,40,41]. In contrast to immunosuppressive drugs, sPIF reduces K+ flux by binding to Kv1.3b potassium channels (via the cortisone binding site) without affecting Ca++ flux in T-cells [42]. Consistent with its immunomodulatory function, sPIF reversed and prevented paralysis and restored myelination by inhibiting neuroinflammation in murine models of experimental autoimmune encephalomyelitis [39,40]. Furthermore, we showed that sPIF promotes myelination and induces oligodendrocyte differentiation by modulating H19 [43]. H19 inhibits S-adenosylhomocysteine hydrolase (SAHH) and induces expression of the nuclear receptor co-repressor 2 (NCOR2) via DNA methylation [43]. In turn, NCOR2 orchestrates oligodendrocyte differentiation, contributing to proper myelination [43]. sPIF’s neuroprotective property was further underscored by its ability to mitigate neuronal loss and microglial activation in a murine model of immature brain injury [44,45]. The neuroprotective effects were attributed, at least in part, to sPIF’s ability to downregulate microRNA let-7 and activate PKA/PKC signaling in the injured brains [44]. In our preterm brain injury model, sPIF treatment enhanced global phosphorylation levels after injury. Specifically, PKA/PKC substrates that are neuroprotective in their phosphorylated state showed enhanced phosphorylation after sPIF administration (namely, Bcl-associated death protein, BadSer112; growth-associated protein-43, Gap-43Ser41; and cAMP-responsive element-binding protein, CREBSer133), and the expression of downstream neuroprotective genes (Bcl2, Bdnf, and Gap43) increased [46]. These sPIF-mediated PKA/PKC mechanisms were TLR4-dependent, as shown by knockdown experiments conducted using a neuronal cell line [46]. sPIF has also been shown to prevent Retinitis Pigmentosa and type 2 diabetes [47,48]. Additionally, recently published data demonstrate that sPIF can cross the blood–brain barrier (BBB) and thus function in the brain [44,46]. In line with the beneficial effects on neuroprotection, perinatal treatment with sPIF was associated with an improvement in working memory in adult Dp(16)1Yey mice (a model of Down’s syndrome (DS)), suggesting that perinatal treatment with sPIF might be an option for mitigating cognitive impairments in people with DS [49,50]. Overall, our sPIF data show strong neuroprotective effects that occur via suppression of neuroinflammation and neuronal death while promoting neuronal plasticity.

Unsurprisingly, sPIF has successfully been tested in radiation models as well. Short-term administration of sPIF protects against GvHD development following lethal TBI (10 Gy) and, in the long-term, in semi-allogeneic and allogeneic (HSCT) settings [51,52]. The protective effects of sPIF include reduced long-term 6-fold mortality, prevention of colonic and skin ulceration, and reduced production of liver, systemic, and endometrial inflammatory cytokines [38,53]. sPIF can also lead to >90% engraftment, increases in systemic immune cells, and bone marrow recovery [54]. The beneficial graft-versus-leukemia effect persists, alongside protection against deleterious GvHD manifestations, suggesting preservation of immune cell function. sPIF administered for 2 weeks to C57BL/6 male/female mice starting 2 h after lethal TBI (8 Gy) led to 100% survival by day 29; in comparison, survival was 0% in controls (*p* < 0.0001) 2 weeks post-treatment. sPIF also partially preserved hematologic profiles, with levels comparable to the baseline [54]. Moreover, in a sublethal GI-ARS model, short-term sPIF restored colon architecture and reduced small-bowel and systemic inflammation [54,55,56]. sPIF is safe and non-toxic, as shown by FDA-directed chronic toxicology studies and an FDA Fast-Track designated safety clinical trial conducted on adult nonpregnant patients with liver disease (NCT 02239562) [57].

Overall, sPIF is an attractive therapeutic agent for comprehensively addressing the multi-organ effects of lethal irradiation. In this study, we aimed to evaluate sPIF’s utility with respect to progressing toward the FDA’s “Animal Rule” [23]. We tested the effect of sPIF (24 h after exposure) in a clinically relevant murine LD50/30 model to determine whether it could improve survival by 4 weeks post-therapy. We evaluated the effect of sPIF on survival, weight recovery, and clinical indices to assess its impact on diverse, multi-organ injuries. We report that short-term administration of sPIF is a safe and effective method for mitigating lethal ARS in the long term.

## 2. Results

### 2.1. Radiation Dose–Response Determination

**Figure 1:**
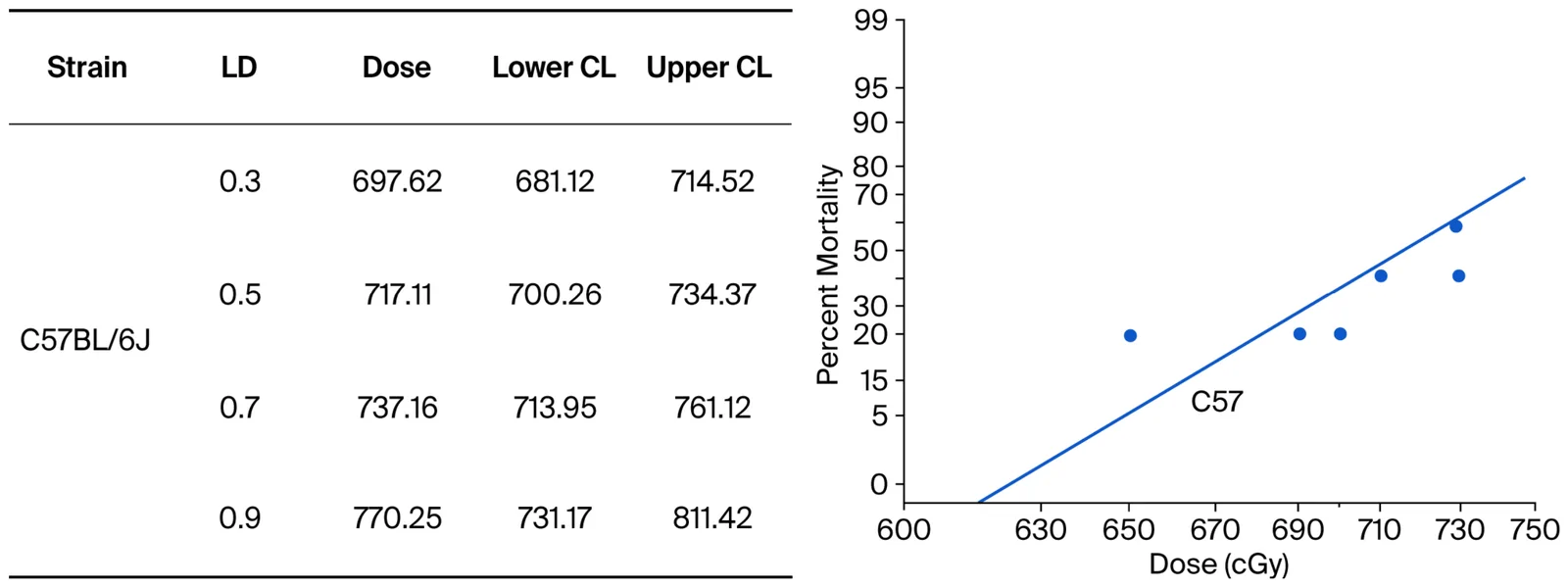
Determination of the lethal dose by testing the survival curve following exposure of two strains of mice to radiation. The LD50/30 dose was determined for the C57BL/6J strain and used for the experiments. Mortality for the C3H/HeN strain was high at low doses of irradiation, so it was not used. The radiation doses in the different treatment groups were determined by using N = 12 male mice with 95% upper and lower CIs. The data demonstrate low variance among the samples tested.

Immediately before the start of this study, we generated a dose–response curve with respect to 104 C57BL/6J mice exposed to TBI equal to 5–9.5 Gy for 30 days, using the same facility employed in the following experiments. All mice survived at 5–6 Gy, while all mice succumbed to levels of 7.5 Gy and higher (these levels were applied to verify the adequacy of the radiation doses). A characteristic steep mortality curve was generated, with LD50/30 (7.17 Gy), LD70/30 (7.36 Gy), and LD90/30 (7.52 Gy) doses estimated. Since very few mice survived the LD70 and LD90 doses, the expected LD50 was used for this study. Notably, this original lethality curve corresponds to mice that were not further handled post-radiation and were followed for only 30 days (Figure 1).

### 2.2. Survival Outcomes in the sPIF, scrPIF, and PBS Treatment Groups

To approximate the clinical management of radiation-incident victims, continuous monitoring, daily weighing, SC injections, and phlebotomy were performed. Also, the follow-up period was increased: up to 41 days instead of 30. Three sets of experiments were carried out using the 7.17 Gy dose. In each set, a survival analysis was extended for 27 days after the last injection (see Table 1). In each independent experiment, the survival rate in the sPIF treatment group was higher than that in the control group. This study was carried out without bone marrow shielding, and the mice were administered solely antibiotics for the first 14 days, a standard supportive therapy used clinically.

In experiment I, BID administration of 1.5 mg/kg sPIF for 14 days resulted in the survival of 75% (6/8) of the mice observed on day 41. Owing to hematoma formation at rotating injection sites in the groups administered sPIF as opposed to the sham control (1.5 mg/kg scrPIF), a single daily injection was substituted for all future experiments. In experiment II, a single daily SC injection of 1.5 mg/kg of sPIF or 1.5 mg/kg of scrPIF was administered for 14 days. In both groups, a hematoma developed at the injection site despite rotation of the injection site. Nevertheless, survival was 23% in the sPIF treatment group, while it was 0% in the sham group. Although a trend toward reduced mortality with sPIF was observed, it did not reach statistical significance. In the final study, the duration of daily injections for both the sPIF and control groups was reduced from 14 days to 10 days, thereby minimizing hematoma formation.

### 2.3. Final Pivotal Efficacy Study: sPIF Is Effective in Reducing Mortality

In this key experiment, a large number of mice were used to compare 1.5 mg/kg sPIF sc. with two control groups, namely, 1.5 mg/kg scrPIF sc. and PBS, the latter of which served as the vehicle. Although we expected the LD50/30 of the sham treatment to be 7.17 Gy, we observed an actual survival of LD85/30. In the sPIF treatment group, 16/39 (41.3%) mice survived, whereas 3/15 (20%) mice survived in the scrPIF group, and only 1/15 (6.6%) survived in the PBS group. All were analyzed on day 37, which was the end of the study, 27 days after the last injection. In the combined sham groups, 4/30 (13.3%) mice showed increased survival when treated with sPIF, representing a 3.1-fold increase. Chi-square analysis showed significant differences in survival between the sPIF group and controls, with a high degree of freedom (Df): 6.96 (*p* < 0.030). Compared to the sham groups, the higher Df of 5.6 (*p* < 0.017) underscores the robustness of the results. This finding indicates that short-term sPIF significantly reduces mortality within 4 weeks post-therapy.

### 2.4. sPIF Monotherapy Totally Prevents Demise for the First 10 Days

Additionally, in the key efficacy study, survival through the final day of injections (day 10) in the sPIF treatment group was 100%, while it was 80% in the sham treatment group (*p* = 0.0034, Df = 8.5, highly significant). Furthermore, 13 days after treatment was stopped (day 23), 50% of the sPIF-treated mice were still alive, whereas only 18% of the sham group survived. The sham group reached 50% survival on day 19 before rapidly declining to 18% by day 23. Therefore, sPIF treatment was also associated with a 21% increase in average survival time, suggesting that sPIF monotherapy can completely prevent mice from dying during the first 10 days of treatment. This finding is critical, as it means victims can be safely evacuated to advanced-care sites, even if the evacuation is significantly delayed.

### 2.5. Combined-Experiment Lifetable Analysis Confirms sPIF’s Efficacy

**Figure 2:**
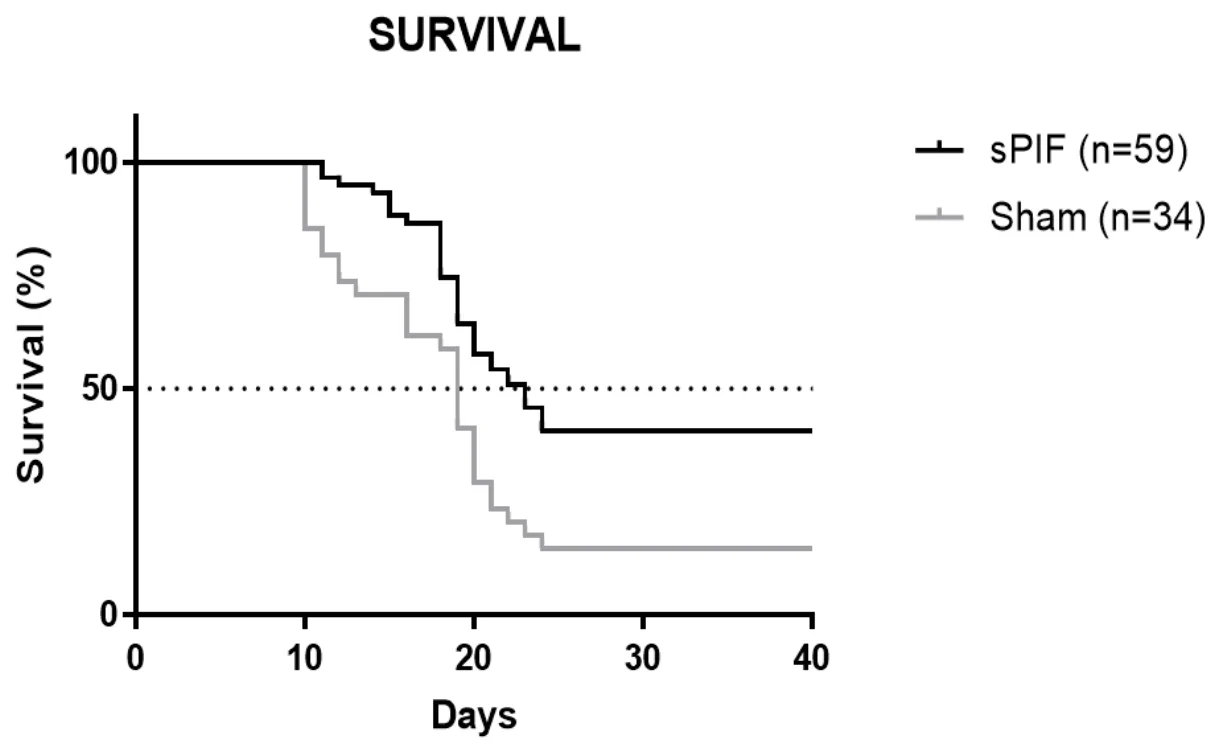
sPIF enhances survival after radiation exposure. All sPIF-treated mice survived for 10 days, while only 80% survived in the control group (*p* = 0.0034, df = 8.5). The survival curves, combining results from all experiments, show that sPIF treatment delays the decline in survival. The Kaplan–Meier curves reveal a slower decrease in survival for the sPIF group (50% survival at day 23) relative to the sham group (50% survival at day 19). Notably, the 50% survival time for the sPIF-treated mice was prolonged to 22 days, whereas it was 20 days in the control group. A log-rank test was used to compare survival curves among treatment groups. Overall survival on day 40 was higher following sPIF treatment relative to the sham (*p* = 0.0036). All mice alive on day 30 continued to survive through the end of the experiment. The dashed line marks 50% survival.

The second-level analysis further validated sPIF’s effectiveness when all three experiments were combined. Only 5 out of 34 (14.7%) mice survived in the combined sham treatment groups. In comparison, 24 out of 59 (40.7%) mice survived in the sPIF group, representing a 2.76-fold increase (*p* = 0.0036). This further supports the findings from the pivotal study (see Figure 2).

### 2.6. sPIF Increases Weight Above the Baseline

**Figure 3:**
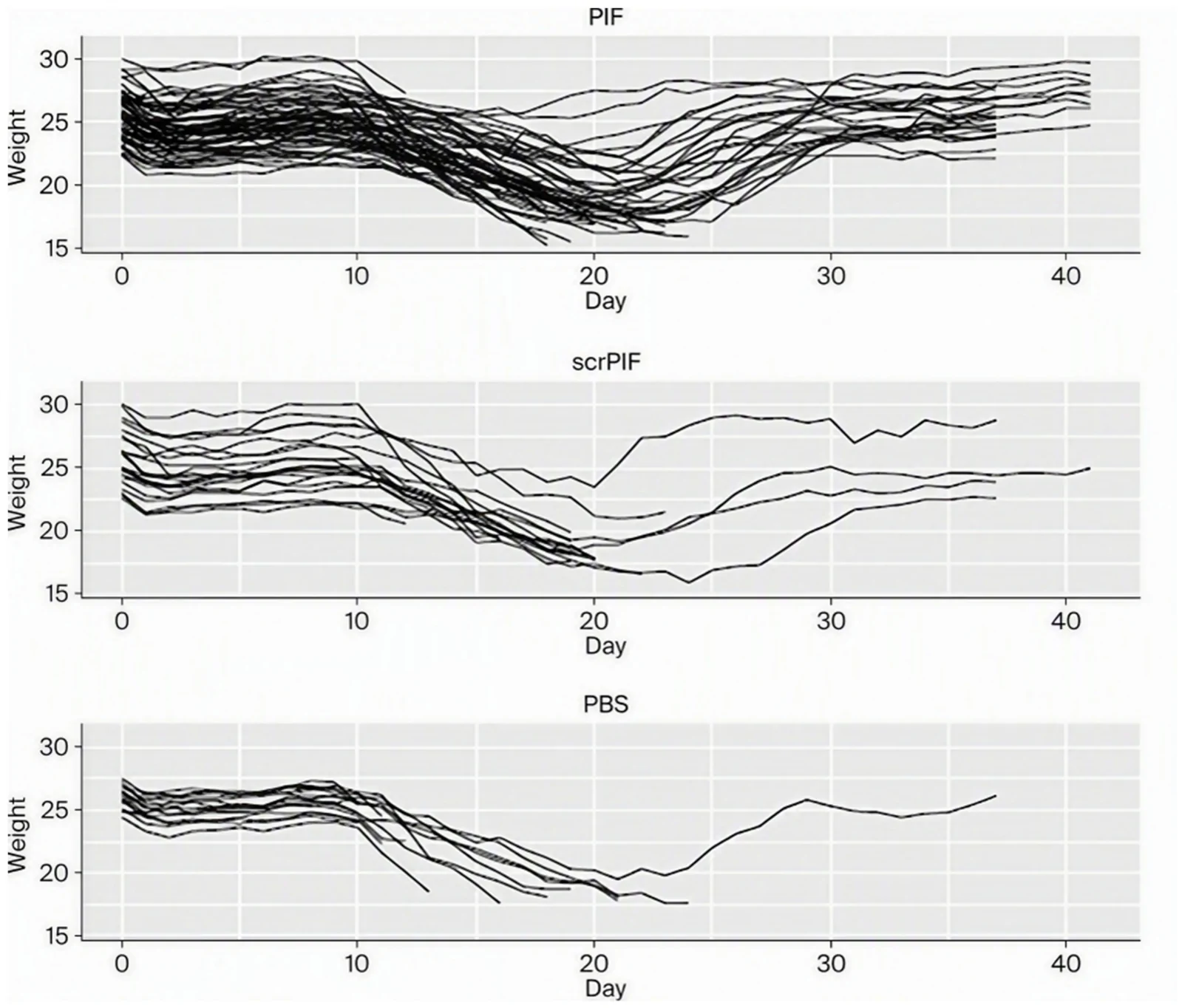
sPIF’s protective effect on mouse weight compared to the baseline. Daily weights are shown for irradiated mice that received SC injections of sPIF (top panel), scrPIF (middle panel), or PBS (bottom panel) for 10 days and were followed up for up to 41 days. Each line represents an individual mouse. The data show that the sPIF-treated mice delayed weight loss for up to 10 days post-treatment. Additionally, after sPIF treatment, the mice fully recovered or exceeded their initial bodyweights by an average of approximately 12% relative to the baseline. In contrast, in the sham groups, none of the mice gained weight relative to the baseline (i.e., exceeded the baseline), and only one mouse returned to its baseline weight.

This study involved male mice approximately 9–10 weeks old, which were expected to become heavier over time in accordance with their natural growth pattern. Because the study lasted up to 41 days, it provided a clear basis for assessing whether the surviving mice could recover and, if so, resume their normal developmental trajectories. An independent toxicological study found that in non-irradiated young male mice (N = 15), the observed weight increase after 40 days was about 12%. This served as a benchmark for the anticipated growth spurt in this study. Individual bodyweights were analyzed across the treatment groups, with baseline values compared to those at the end of the study. The mean weights at the start of the study (pre-radiation) were similar across groups (*p* = 0.2). Figure 3 shows individual weights over time for each of the three groups (sPIF, scrPIF, and PBS control).

The greatest weight loss in the sPIF treatment group was reached only by day 25, more than 10 days after sPIF administration was stopped, and this was followed by a rapid recovery to above-baseline levels. The sPIF-treated mice that survived for up to 4 weeks after drug treatment mostly recovered to weights above their baseline. On average, this weight increase was 10–15%. Weight recovery was significant until day 37 relative to the baseline (*p* < 0.05). In contrast, only one of the five mice in the control group returned to its baseline weight. Additionally, this mouse continued to lag in terms of weight gain, contrary to expectations. This indicates that the surviving sPIF-treated mice followed their natural growth patterns despite the severe ARS.

### 2.7. sPIF Partially Protects Hematologic Profiles

The 7.17 Gy TBI dose used in this study induced significant H-ARS. This result was expected because this was an LD85/30 study, and no bone marrow shielding was used. In the three treatment groups, all blood parameters (hemoglobin [Hgb] level, hematocrit [Hct] percentage, platelet count, and total WBC count) decreased by day 4 and reached a nadir on day 10. Platelets and hemoglobin recovered first, while WBCs continued to lag. No significant differences in the time course of these changes were observed among the three treatment groups.

**Figure 4:**
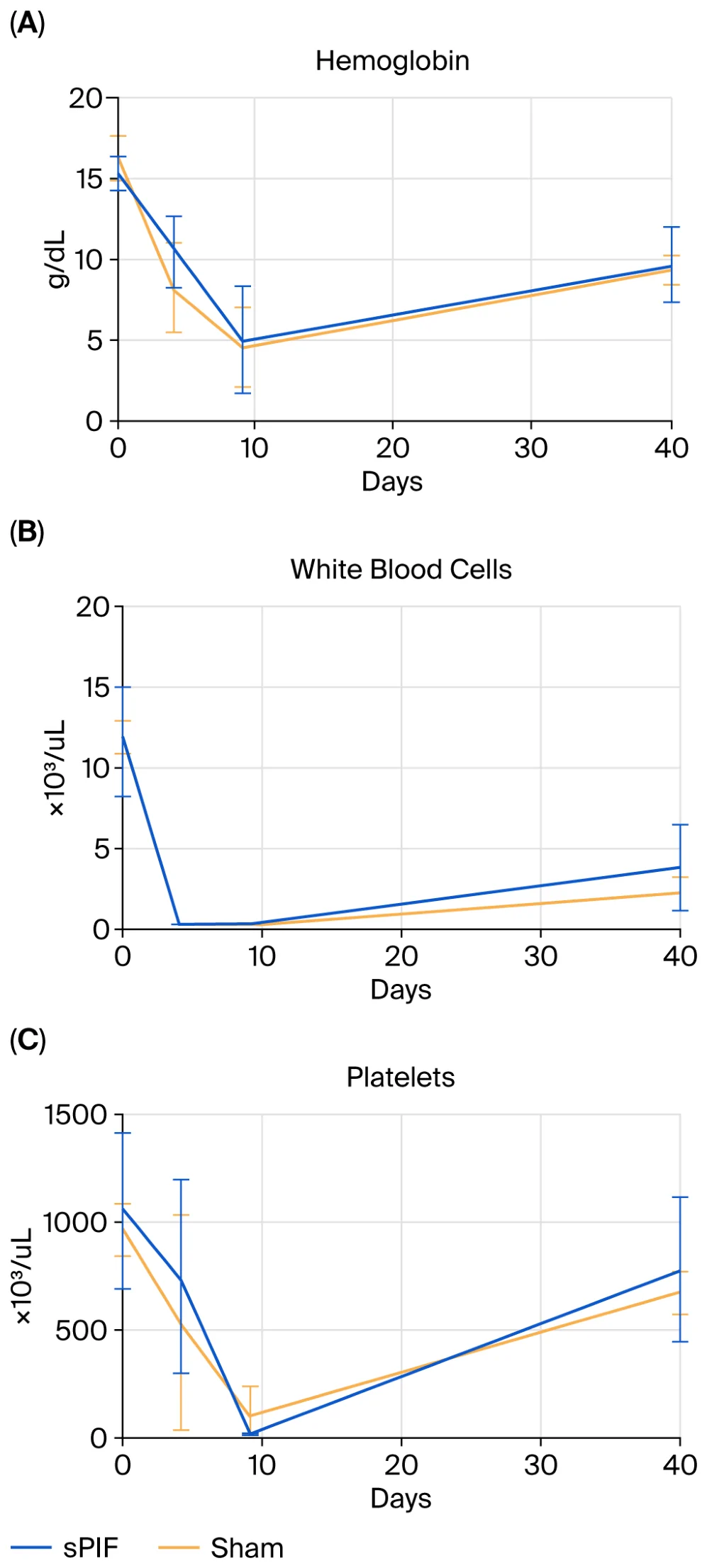
Hematological parameters of the experimental mice. Mean ± SEM levels on days 0, 4, 8, and 40 for (**A**) hemoglobin levels, (**B**) WBC counts, and (**C**) platelet counts in mice treated with either SPIF or scrPIF (sham) are shown. There were no significant differences in levels between the two groups at any timepoint. Day 0 = day of radiation exposure.

Since there were 24 survivors in the sPIF group and only 5 survivors in the combined scrPIF and PBS groups, comparative analysis of the hematologic results was limited. Additionally, only 4 of 30 control mice survived until day 37 in the final study, and because of their poor condition, few data could be collected owing to blood clotting in the collection tubes. This limited our ability to analyze this group further relative to the larger number of animals (N = 16) available after sPIF treatment in the final study. Therefore, the data are reliable only for early timepoints up to day 10, during which no significant differences in blood parameters were observed. Overall, *p*-values of 0.0694 (Hgb levels), 0.6953 (platelet counts), and 0.2690 (WBC counts) were calculated regarding differences between the treatment and sham (Figure 4). In a secondary analysis, individual Hgb levels, platelet counts, and WBC counts were plotted and compared directly to baseline values. Given that individual values among the sPIF-treated mice varied—as expected—those with data available from the baseline measurement through the end of the study were further analyzed.

When comparing post-sPIF treatment values with pretreatment values, we observed a moderate decrease in HCT in nine mice initially tested. Compared to the values at the end of the study, the decline ranged from 5% to 22% (mean +/− SD: 13.7 +/− 6.7%). These changes are clinically insignificant, indicating only mild anemia. Additionally, three mice had no pretreatment levels available, with HCTs of 28, 23, and 15%. In the irradiated, untreated mice (N = 3), despite not being injected or included in the final study, the %HCT decreased in all three mice. This suggests partial sPIF-induced protection. Regarding platelet counts, no definitive conclusions could be drawn owing to the limited number of samples; among those available, a mild decrease of 15–20% was observed. In the sPIF-treated mice, a significant decrease in WBC count amounting to 65–70% was noted. In irradiated but unmanipulated mice, there was also evidence of H-ARS. Supplementary Figure S1 depicts individual results (hemoglobin, white blood cells, and platelets) among the three treatment groups (sPIF, scrPIF, and PBS) and in mice that were not treated (those that did not participate in the final study).

### 2.8. Resolution of Hematomas Post-sPIF Administration

At the necropsy on days 8 or 10, hematomas were observed at the injection sites in most mice, irrespective of treatment. Consequently, total injection time and number were limited to once daily for 10 days. In the surviving mice, the hematomas were resolved, and there was no evidence of bleeding. The survivors’ extremities and bones appeared grossly normal by the end of the experiments. Overall, despite the IR-induced effect on the hematologic profile, sPIF administration remarkably led to hematoma resolution and prevented bleeding or sepsis by 4 w post-therapy.

### 2.9. sPIF Improved Spleen Histology

**Figure 5:**
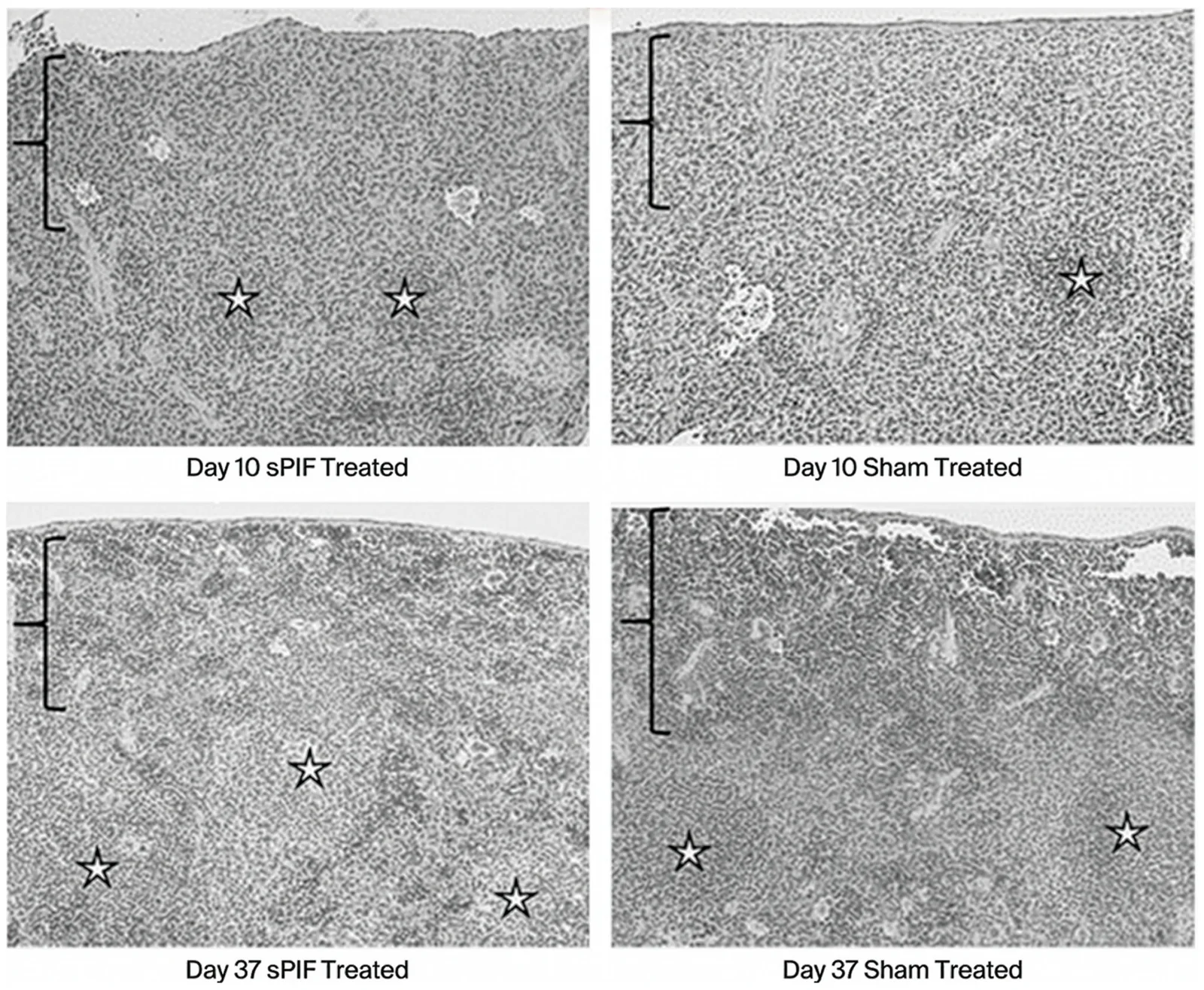
Spleen histology. Representative histological images at 10× magnification. Both the sPIF- and sham-treated mice showed depletion of white pulp (stars) and red pulp (left braces) on day 10, with similar findings on day 4. By day 37, reconstitution of white pulp and expansion of red pulp with hematopoietic elements were evident in the sPIF-treated mice, whereas the sham-treated mice showed evidence of surface necrosis. Hematoxylin and eosin staining was used.

Spleen size was reduced in all irradiated animals as early as the first necropsy (day 4), and this trend persisted through day 10. The splenic regions with lymph nodes (white pulp) in the sPIF-treated mice were better preserved on days 7 and 37 at the end of the study compared with the control (Figure 5). This is shown by the increased number of areas with lymph nodes (white pulp) and the lack of capsule necrosis compared to the sham-treated control.

### 2.10. sPIF Therapy Improves Colon Histology

**Figure 6:**
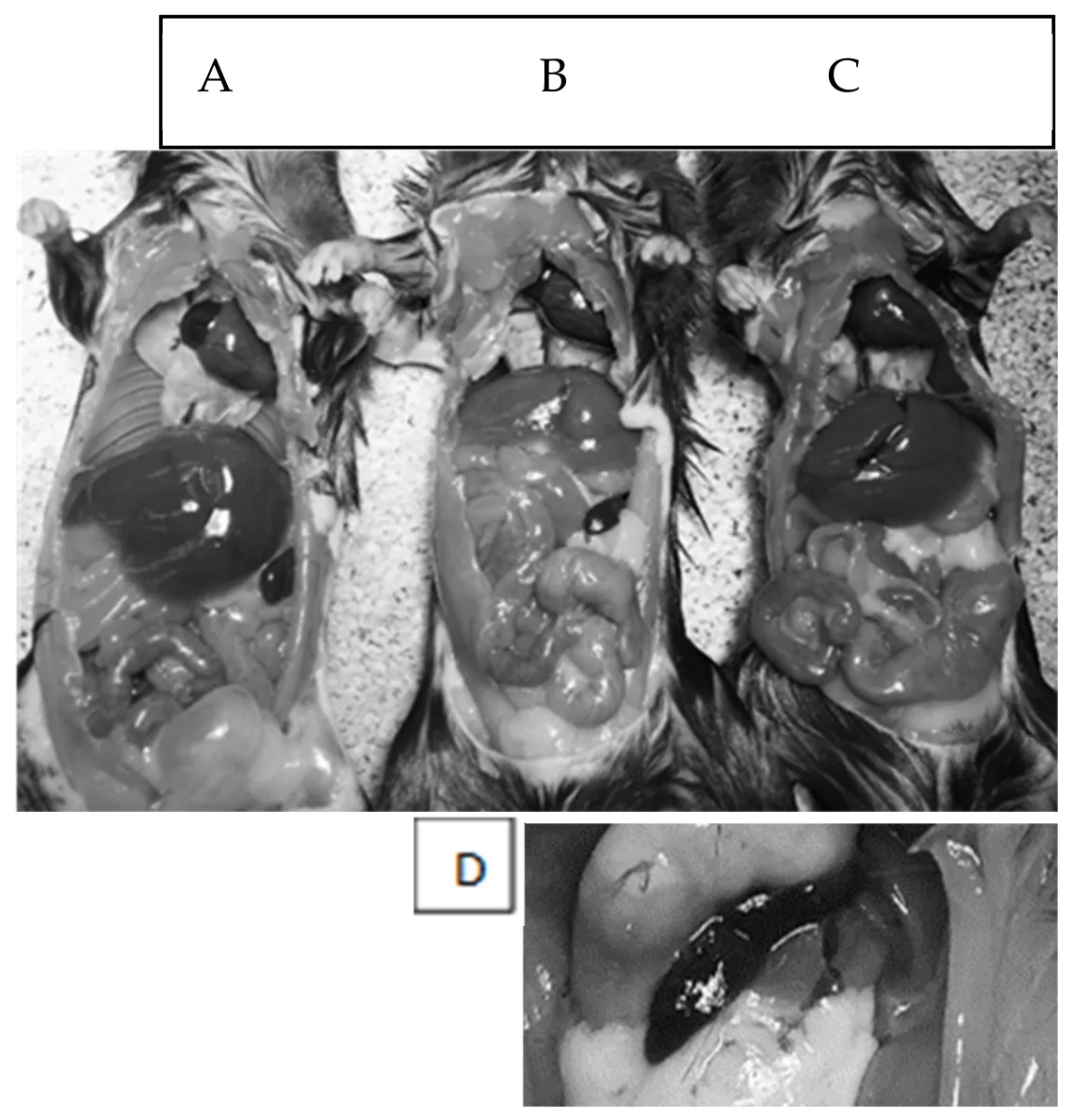
Day 8 necropsy. Representative mice from each group: (**A**) control (untreated), (**B**) sPIF-treated, and (**C**) sham-treated. Note that both (**B**) and (**C**) show mild distention of the bowel/cecum. Additionally, the spleen was smaller after the sham treatment ((**D**); at higher magnification) compared to the spleen in the control (**A**) or sPIF-treated (**B**) mice.

**Figure 7:**
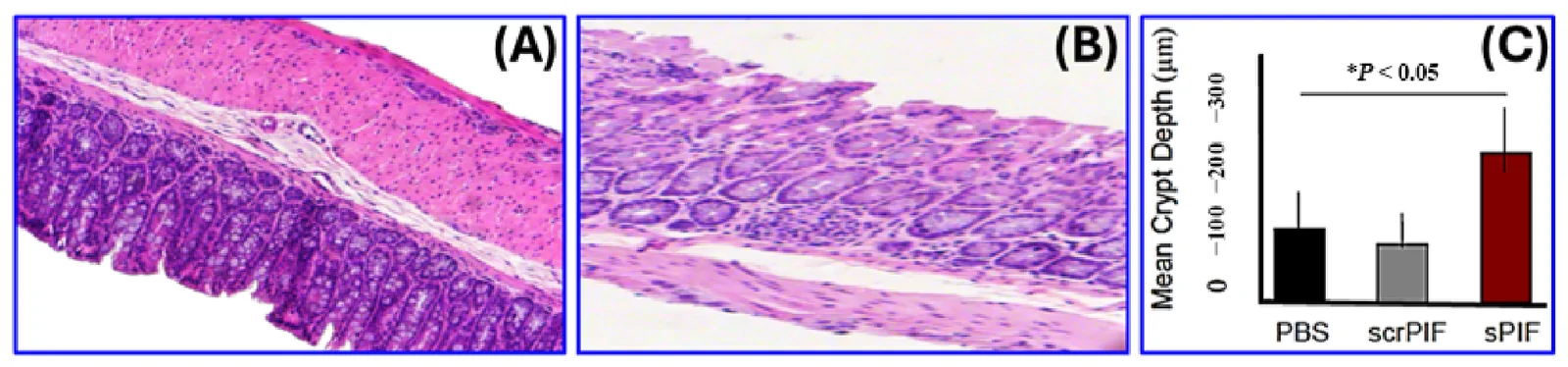
Colon histology. Representative images of mice (**A**) treated with sPIF and (**B**) the sham. sPIF improved colon histology by restoring colon crypts and the basal membrane on day 41 of the study. (**C**) sPIF enhances crypt depth. ANOVA, *N* = 75+/−5/group. * *p* < 0.05 versus PBS and scrPIF Error bars represent means ± SEMs.

The images in Figure 6A–C show representative colon images of the naïve, sPIF-treated, and sham-treated mice. Compared to the naïve controls, the colons in the treated groups appeared moderately distended with soft, custard-like fecal material (occasionally forming pellets), as observed at the 10-day necropsy. The size of the colon returned to normal by the end of the study. No diarrhea was noted at any point during the study. This suggests recovery of the GI tract. When examining the colon histology section on day 41, we noted that crypt depth was greater in the sPIF-treated animals relative to the sham-treated ones (*p* < 0.05). (Figure 7). On day 4 of the study, there was evidence of significant cell death. Using the TUNEL assay in the colon, we demonstrated that, compared to the protective action of sPIF, the colons of the scrPIF-treated mice had undergone significant apoptosis (2.7+/−0.3 vs. sPIF 0.3+/−0.3 *p* < 0.05).

## 3. Discussion

Lethal total-body irradiation (TBI) involves high-intensity exposure, causing widespread, severe damage, ranging from acute to chronic, hematopoietic (H), gastrointestinal (GI), neurovascular, and/or cutaneous and vascular damage that develops after exposure to ionizing radiation (IR). In addition to long-term survival, both hematopoietic (H-ARS) and gastrointestinal (GI-ARS) acute radiation syndrome are addressed herein.

Acute radiation syndrome (ARS) results from voluntary and/or involuntary exposure to radiation in military/terror-related and civilian populations and can occur in densely populated or remote settings and affect healthy populations and those with pre-existing medical conditions. A “universal” safe, easy-to-administer countermeasure is currently an unmet need.

Synthetic premplantation factor (sPIF) replicates the endogenous PIF features and therefore possesses the features necessary for comprehensively addressing the spectrum of radiation-induced disease. sPIF has been shown to address the root cause of damage, protecting against oxidative stress and cellular damage through targeted receptor-dependent action [28,38,39,40,41]. Overall, sPIF constitutes a back-to-nature approach to safely and effectively addressing cellular/organ damage both locally and systemically [39,40,41,42,51,52,54,57].

In our study, we observed 100% survival at 10 days in the sPIF-treated mice vs. only 80% in the control/sham group. First and foremost, we addressed the stated main goal, showing that sPIF improves long-term survival rates in lethal TBI ARS. Additionally, sPIF increased both 50% survival time and overall survival time until the end of the study. Beyond improved survival, this study demonstrates that sPIF addresses hematologic profiles (H-ARS), thereby preventing fever and sepsis. Notably, sPIF totally restores weight and improves the all-important gastrointestinal architecture and function (GI-ARS). 

sPIF improves GI architecture and function and restores weight and colon crypts. In tandem with a partially restored hematological profile, one of the most important hurdles to overcome is radiation-induced damage when radiation causes GI damage. Without properly functioning GI architecture and activity, the irradiated subject “wastes away,” unable to feed/process nutrition, resulting in an imbalanced immune system. An important indicator of well-being in these mice is stable weight and a sustained growth spurt. Though this model was not directly geared toward assessing GI-ARS per se (it was not a standard GI-ARS model), sPIF showed definite protection in the model used, which does not involve the use of bone marrow shielding.

Furthermore, sPIF was observed to prevent weight loss for up to two weeks after the end of treatment and, importantly, even resulted in an average weight gain of about 12% with respect to the baseline, supporting the expected normal growth pattern (NIH/NCATdBrIDGs) toxicology study data. The sham-treated mice mostly died, and the few survivors’ weights failed to recover, suggesting that sPIF can help overcome heightened stress and enhance food intake. This weight recovery is due to the restored colon crypt architecture—which also persisted after the end of the sPIF treatment—and the lack of diarrhea, thus preventing malnutrition in the long term. The TUNEL data also demonstrated reduced early IR-induced colon cell apoptosis. Though this model does not involve bone marrow protection, these findings reflect potential protection against GI-ARS, constituting a current unmet need. sPIF’s protective effect aligns the findings of with our previous study, which detailed sPIF’s protective role in the GI tract, including restoration of colon architecture and function, with recovery of the basal membrane after IR 6Gy and sPIF treatment noted within 1–2 days over a 3-day period [54].

To pinpoint sPIF’s GI-protective/regenerative mechanisms, detailed jejunum analysis using genomic and pathway methods was conducted, revealing sPIF’s involvement in protein–RNA interactions, mitochondrial oxidative pathways, and responses to cellular stress [54]. Moreover, sPIF reduced inducible nitric oxide synthase (iNOS) levels and increased the expression of protective factors such as B7h1, demonstrating targeted protection, along with decreased systemic inflammatory cytokine levels [54]. This integrated (local and systemic) protective effect (beyond ARS) of sPIF is also observed in several immune disorders, where additional cellular protective and regenerative mechanisms have been identified [42,51,52].

Short-term sPIF administration was found to lead to long-term effects (relevant ARS model). First, a necessary standard H-ARS LD50/30 curve was generated (Figure 1) without further manipulation. For the actual study, mice were observed for up to 41 days, whereas there was a 30-day period in the LD curve study. This approach was taken because the sPIF-treated mice continued to survive beyond the standard 30-day window, providing valuable additional data that demonstrate long-term efficacy. The results showed that sPIF’s action/effect following short-term administration leads to prolonged survival, lasting up to 4 weeks post-therapy, thereby allowing a significant delay in access to care.

Notably, in earlier experiments, despite site rotation, BID SC sPIF injections in all treatment groups caused hematoma formation at the injection site by days 4–8 due to low Hb and platelet counts, followed by slow recovery. Switching to a single SC sPIF injection over 14 days produced a similar effect, whereas a 10-day injection resulted in only a temporary hematoma that resolved completely by day 37 in the sPIF-treated mice. Compared with the baseline, a mild decrease in Hb was observed at the end of the study, while platelet data remained incomplete; importantly, however, this enabled long-term recovery in the surviving mice. In the sPIF treatment group, the baseline WBC count was significantly lower than that at the end of the study. Nevertheless, antibiotics were administered (for only 14 days), and the study continued for an additional four weeks. The lack of fever or sepsis observed indicates at least partial preservation of immune protection. This may be further supported by the apparent improvement in spleen architecture, with more white pulp foci without necrosis relative to the sham. Notably, the irradiated untreated mice (N = 3), despite not receiving injections or being included in the final study, also showed a decrease in %HCT. This finding indicates that even without intervention, an adverse effect on the hematologic profile was evident, and sPIF may offer partial protection in this regard. This observation warrants further investigation.

The radiation dose rate used in this study was 1.27 Gy/min, which is likely higher than previously used rates (see Materials and Methods), a fact that could have further increased overall lethality. In the efficacy study, sham treatment resulted in only 4 of 30 mice surviving, reflecting the severity of the dose. In contrast, despite the high lethality, sPIF increased mouse survival to 16 out of 39, an approximately three-fold increase, and restored weight and colon architecture.

In this paragraph, we provide context regarding the sPIF model used and confirm the results of a previous ARS-related study. In this study, we used an sPIF dose (1.5 mg/kg) that was shown to be effective in a previous (LD100/30) H-ARS study, which involved radiation without bone marrow shielding. The main difference was that sPIF was administered preventively, i.e., 2 h after irradiation, rather than as a treatment, a scenario in which access to therapy might be delayed. We also used scrPIF (with the same amino acids as sPIF but in a different order) as an important control; it was not used as a control in the previous study. The data reported in the previous H-ARS study showed that in both male and female mice, sPIF administered for 14 days prevented mortality in 100% of mice, including at 2 weeks post-treatment, whereas the figure was 0% among the controls. Furthermore, sPIF fully restored hematologic profiles (HCT, WBC, and platelets) to the baseline, including several immune phenotypes [54]. In addition, the immune profile and femur bone marrow recovery were confirmed 24 h after sublethal TBI H-ARS (6 Gy). Since no sex differences were observed in regard to the protective effect of sPIF in the previous study, herein, only male mice were studied in large numbers, improving the study’s power.

Although the lethal mouse model of H-ARS mimics the progression of acute radiation injury in humans, there are differences in the timeline of development and medical management of the syndrome. When evaluating this study’s promising results, it is important to consider the limited basic life support given to mice relative to the detailed support measures that are standard in human medical care. For example, in humans, a single intravenous access site would suffice for infusions of fluids, electrolytes, nutrients, and blood products, but such sites are not readily available in field conditions. However, if feasible, such an access site could reduce the need for daily SC sPIF injections since it could be administered via infusion and would eliminate the need to weigh the subjects, a practice required for mice but not for humans.

Whereas several current therapeutic approaches are aimed at addressing hematologic repair, only a few FDA-approved LGFs, namely, Neupogen^®^, Neulasta^®^, and Leukine^®^, along with related similar analogs, predominantly address WBC neutrophil recovery; in addition, they also lead to thrombocytopenia. Hence, they have harsh, life-long side effects.

As previously reported in a series of studies evaluating the effect of sPIF in GVHD, this particularly auspicious model closely related to ARS involves TBI and sPIF’s ability to decrease mortality, promote transplant acceptance, control cell/organ growth, and, in general, improve immune homeostasis. In these studies, sPIF was shown to diminish deleterious GVHD (acute and chronic) while maintaining the beneficial graft-versus-leukemia (GVL) effect. In a semi-allogeneic bone marrow transplant model, administration of sPIF for 14 days 24h after exposure to lethal radiation, with up to 4 months of follow-up, reduced mortality by over 80% and improved engraftment. sPIF also protected the skin, liver, and colon while reducing systemic inflammation, supporting sPIF’s suitability as a therapeutic candidate for the prevention of GVHD [42,51,52,54].

This study’s findings may accelerate the FDA’s approval of sPIF for administration to patients, directly creating an impetus for using sPIF in a clinical setting via applying for approval for use in treating ARS via the “Animal Rule,” although this is outside the scope of this study. sPIF’s unique level of safety/tolerability has been confirmed at two levels: in a First-in-Human, FDA-approved, fast-track-awarded clinical trial (NCT 02239562), and in a comprehensive FDA-guided, long-term safety toxicology/toxicokinetic study, which yielded No Observed Adverse Effect Level (NOAEL) (NIH/NCATdBrIDGs).

First-in-Human status: sPIF is safe and does not trigger any deleterious drug-to-drug interactions. In a First-in-Human, FDA-Fast-Track-approved Phase I clinical trial, sPIF exhibited no adverse drug-to-drug interactions (with respect to steroids/immunosuppressive drugs and others). Accordingly, sPIF administration is unlikely to interfere with the various medications that radiation victims might already be taking [57]. The clinical study was performed on patients with autoimmune disease (not human volunteers). This study is relevant to our study, as it demonstrates sPIF-mediated immune protection and involves the liver, which is directly affected by radiation.

sPIF is currently formulated as a human-grade cGMP-ready-to-inject solution that remains stable at high temperatures without loss of activity and is readily SC-injectable, making it suitable for remote site storage and field use [51,52,54]. Thus, safe sPIF monotherapy (which can also be self-injected) could completely prevent mortality, even with a major delay of up to 10 days in evacuation and access to additional supportive measures. The drug’s documented safety enables sPIF administration to IR victims irrespective of their medical condition and the medications that they take. This sPIF advances H-ARS and related terror/military and, in parallel, civilian lethal TBI damage acute/delayed management, including GI-ARS.

This study’s strength lies in our use of a combat-realistic, standardized lethal model that focuses on the lethal H-ARS LD85/40 and provides additional support for GI-ARS in realistic settings not involving bone marrow shields. Limiting the duration of sPIF administration in the key efficacy study to 4 days led to a 4-week post-therapy follow-up with minimal hematomas. We also confirmed that the sPIF dose and duration demonstrated in the previous H-ARS preventive study are effective. Lastly, using a scrambled peptide (scrPIF) as a control revealed that the sPIF configuration is specific and that its action depends on targeted receptor interaction, which is not replicated by a peptide of the same size and amino acids in a different order. This study corroborates and strengthens similar studies, which showed increased survival, restoration of hematologic profiles, restoration of GI architecture and function, and, in general, sPIF’s ability to safely restore homeostatic immunity.

This study’s limitations include the high sham-group mortality, which, at the end of the study, limited analysis of blood and histological samples even though they clearly indicated that there was an around 3-fold decrease in mortality with the sPIF treatment. Consequently, a comparison was only possible of sPIF’s effect at baseline with that at the end of the study. Additionally, only male mice were studied, unlike in the LD100/30 preventive study, which included both sexes across two species. As noted in this study, overall mortality in the control-PBS and scrambled sPIF-treated groups was very high (14.7% (5/34) to 40.7% (24/59); *p* = 0.0036), whereas sPIF increased survival ~3-fold at the end of the study in the 85%-mortality-rate study. Such high mortality at the end of the study clearly limited the number of cases in which comparisons between sPIF-treated and control blood indices could be made. Therefore, when WBC, RBC, and platelet measurements were available, comparisons were made between levels at the start and end of the study. Moreover, because of the severe disease, the blood coagulated, which limited further analysis of blood indices.

There was a trend toward lower WBC counts, but spleen activity appears to have been preserved, which may have compensated for the infection risk since sPIF led to protection against infection and sepsis, a key factor in maintaining immune function one month post-therapy. Furthermore, there was only a mild decrease in both RBC and platelet counts, and there was no evidence of bleeding diathesis when SC injections were administered for 10 days. Overall, albeit with limited observations owing to the ARS model’s severity, the data support the view that, beyond the ~3-fold increase in mortality, restored GI also conferred partial H-ARS protection, indicating an integrated sPIF-induced protective effect long after the end of medication administration.

In summary, short-term SC sPIF monotherapy in a lethal H-ARS model of male mice (85/40) increased survival ~3-fold and protected the GI tract, restoring architecture and function by delaying weight loss and restoring weight 4 weeks post-therapy. Based on these promising studies, NIH/NIAID awarded us with a contract to evaluate sPIF in GI-ARS models. Overall, sPIF is a promising, safe, and efficient drug with the potential to significantly mitigate ARS and, in general, radiation damage, eventually in several or all of its clinically realistic manifestations.

## 4. Materials and Methods

### 4.1. Preimplantation Factor

Synthetic PIF (sPIF) (MVRIKPGSANKPSDD) and scrambled PIF peptide (ScrPIF) (random peptide sequence—GRVDPSNKSMPKDIA), used as the sham-control treatment, were produced through solid-phase peptide synthesis (Peptide Synthesizer, Applied Biosystems, Foster City, CA, USA), employing 9-fluorenylmethoxycarbonyl chemistry. Final purification was carried out using reversed-phase HPLC. Peptide identity was confirmed via matrix-assisted laser desorption/ionization time-of-flight mass spectrometry and amino acid analysis, with HPLC purity exceeding 95%, and verified via mass spectrometry at Bio-Synthesis (Lewisville, TX, USA).

### 4.2. Mice

C57BL/6J male mice—9–10 weeks old, from The Jackson Laboratories (Bar Harbor, ME, USA)—were allowed to acclimate for one week in a Yale University AAALAC-accredited facility before use. The mice were kept on ventilated racks with filtered air, an automatic water supply, and sterilized standard mouse chow.

### 4.3. Radiation Procedure

For total-body ɣ-irradiation (TBI) exposure, the mice were placed in a pie setup (Braintree Scientific, Inc., Braintree, MA, USA) in groups of 10 on a 12-inch rotating turntable within a 14 × 14-inch chamber of a 137Cs irradiator (JL Shepard Mark I Irradiator, model 68A; JL Shepard and Associates Inc., San Fernando, CA, USA), for which isodose curves have been determined. The machine’s dosimetry was evaluated prior to exposure.

#### 4.3.1. Radiation: Lethal-Dose (LD) Curve Generation

A dose–response curve was generated using 104 C57BL/6J mice exposed to radiation doses ranging from 5 to 9.5 Gy TBI. Critically, after irradiation, these mice were not injected or handled for blood tests or weight assessments. The LD curve was generated up to day 30. This model was used to create an optimized survival curve with minimal stress relative to the next set of experiments, which involved daily handling, weighing, injections, phlebotomy, and longer observations until day 41 to simulate a more realistic scenario in humans (Supplementary Figure S1).

#### 4.3.2. Experiments Testing sPIF’s Effect Using a Clinically Realistic Model

The same radiation exposure dose was used in all experiments. These studies were designed to closely replicate the clinical management of a radiation victim. On day 0, the mice were exposed to TBI at 7.17 Gy (1.26 Gy/min), resulting in an estimated fivefold-higher cumulative radiation dose 24 h post-exposure. Three sets of experiments with sPIF were conducted. In this case, however, the observation period was extended to day 41 rather than day 30, as used for the original LD curve. Table 1 shows the individual mice studied and sacrificed, along with their survival.

We aimed to replicate a realistic clinical scenario of TBI in which additional protective measures are limited or unavailable, thereby reducing lethality. Therefore, importantly, we did not use any partial bone marrow shields in any of the experiments when testing sPIF’s potential protective effects. Additionally, daily handling and weighing were performed to continuously monitor clinical status.

We used scrPIF in all experiments as a control (a peptide with the same MW as sPIF but in a different amino acid order). The lack of activity of scrPIF, as documented in several in vivo studies, serves as a realistic negative control rather than just a vehicle.

Experiment I: sPIF was injected SC (0.75 mg/kg BID) daily for 14 days and compared with biologically inactive scrPIF (sham, 0.75 mg/kg, injected SC BID) for 14 days. The effects were followed post-therapy for up to 41 days. In this experiment, some mice were removed on days 4, 8, and 10 to generate hematological and histological data.

Experiment II: To reduce hematoma formation caused by BID injections as observed in Experiment I, all groups instead received single daily SC injections, as in Experiment I, with the full daily dose of 1.5 mg/kg or sham PIF (scrPIF—1.5 mg/kg, injected SC in the control) for 14 days. These mice were monitored post-treatment for up to 41 days. In this experiment, 3 mice were not injected, and several mice from the treated groups were removed and sacrificed on days 4 and 8 to collect hematological and histological data.

Experiment III—Pivotal Efficacy Study: Since hematomas still developed after injection for 14 days (Experiment II), injections in all three groups were limited to only 10 days (N = 15–39/group). sPIF was injected SC at 1.5 mg/kg per day for 10 days and compared to scrPIF injected SC at 1.5 mg/kg or PBS injections. These mice were followed post-treatment for up to 37 days. In this study, no mice were sacrificed unless medically necessary, and none were sacrificed until the end of the experiment. This allowed for full cohort analysis involving a large number of mice per group. As such, it is an important complement to using PBS as a vehicle control for comparing the effect of sPIF. The large number of mice also enabled us to conduct a power analysis comparing the three groups.

SC was selected over other routes for the following reasons: (a) SC injections are preferred primarily because they provide a slow, sustained rate of drug absorption. This pharmacokinetic profile accurately mimics the continuous, low-dose delivery often required for biotherapeutics while bypassing the first-pass metabolism associated with oral administration. (b) SC is suitable for mid-sized molecules (like sPIF-15 amino acid peptides). Large or mid-sized biologics, such as synthetic peptides or antibodies, often cannot easily enter local blood capillaries due to their size. They are preferentially absorbed via the lymphatic system, a process that can easily be evaluated using SC administration in animal models. (c) Lastly, this approach minimizes tissue damage and predictive clinical bridging.

#### 4.3.3. Antibiotics

Starting 24 h after radiation, automatic watering ceased for all mice, and they were given water bottles containing enrofloxacin (Baytril, 0.27 mg/mL; Bayer Pharmaceuticals, Whippany, NJ, USA) for 14 days, after which they were returned to automatic watering. The antibiotics were prepared in fresh form every 7 days, as enrofloxacin has been shown to remain stable in drinking water for up to 1 week.

#### 4.3.4. Supportive Therapy

Immediately after irradiation, the mice were returned to their cages, with moist chow placed on the floor along with a water bottle (or automatic watering was provided, as noted above). The mice were monitored, weighed, and provided with fresh, moist chow daily throughout the study.

#### 4.3.5. Sacrifice

Mice were randomly selected (e.g., the third animal in the second cage, prior to weighing) from each group for scheduled sacrifice by CO2 asphyxiation on day 4 and day 8 or 10, when blood and tissue samples were collected. The euthanasia criteria included >25% weight loss or being hunched or scruffy or showing other signs of acute distress. In experiments I and II, mice surviving until the end of the study were sacrificed on day 41. In the pivotal efficacy study (Experiment III), any surviving mice were sacrificed on day 37 (27 days after the last injection).

#### 4.3.6. Necropsy

During the scheduled necropsy, gross anatomy and morphology were examined and documented. Blood was collected via cardiac puncture; serum and EDTA-anticoagulated blood samples were obtained for cell and differential counts. Trans-cardiac perfusion through the left ventricle with heparinized saline (10 U/mL) was performed until the liver appeared blanched. Samples from the spleen, liver, colon and small intestines, skin, and femurs (for bone marrow) were collected during Experiments I and II, on day 41 of Experiment III, and on day 37. These samples were prepared for various analyses: paraffin embedding in 4% paraformaldehyde (PFA), RNAlater (Qiagen, Valencia, CA, USA) for PCR, snap-freezing in OCT for immunohistology, and freezing for protein extraction. The colon was collected, flushed with saline, and prepared as Swiss rolls for fixation in 4% PFA and then transferred to 70% ethanol before embedding for histology. Femurs were fixed, transferred to water, decalcified, and embedded for histology. Note that not all tissues were collected at each timepoint.

#### 4.3.7. Histology

Fixed tissue was embedded in paraffin, and hematoxylin and eosin (H&E) staining was performed for morphological analysis. A board-certified veterinary pathologist examined the slides. Apoptosis (cell death) was assessed using immunohistochemistry for terminal deoxynucleotidyl transferase-mediated deoxyuridine triphosphate nick end labeling (TUNEL) on the colon. Sections were stained with hematoxylin and eosin (N = 3 scrPIF and 16 for sPIF). Histological samples were analyzed in a blinded manner. To determine crypt depth, at least 10 well-oriented, full-length crypt units were counted and averaged for each sample. Measurements were performed using a digital imager. Mean crypt depth was reported in µm.

#### 4.3.8. Weight Assessment

Individual weights were recorded daily to calculate the percentage change in weight with respect to the baseline. Weights were measured until the mice reached a moribund state, at which point they were euthanized. Two types of analysis were performed: one comparing individual weights at the end of the study to the baseline, and another calculating the average weight for all surviving mice in each treatment group, with the percentage change in weight plotted over time.

#### 4.3.9. Blood Collection and Analysis

Mice were weighed and marked on the earlobe for identification before being placed under anesthesia (isoflurane/propylene glycol). A total of 100 µL of blood was collected from the retro-orbital sinus into EDTA collection vials (Safe-T-fill, Ram Scientific, Austell, GA, USA) 1–3 days prior to radiation and at the indicated times up to 37–41 days after radiation exposure. Blood samples were analyzed on a Coulter AcT-diff analyzer (Beckman Coulter, Inc., Brea, CA, USA).

### 4.4. Statistical Analysis

#### 4.4.1. Survival

Due to unexpectedly high mortality (LD85) and the study extending up to 41 days (instead of the commonly used 30), results were combined in the initial analysis to produce more-reliable Kaplan–Meier curves. Survival times were recorded until the mice were either found dead or sacrificed. The log-rank test was used to compare survival curves for treatment groups. In the critical Experiment III, a Chi-square multigroup analysis was conducted, with a significance level set to *p* < 0.05. The average weight of mice at the start of the study was compared to that at the end, with significance also set to *p* < 0.05, similar to the analysis of colon crypt length comparing sPIF with sham-treated mice.

#### 4.4.2. Blood

Due to high mortality, only a limited number of non-coagulated blood samples could be collected in the control group on day 37 of the final study (N = 4). The log-rank test was used to evaluate the effects on various blood indices. Mixed-effects models were used to compare changes in blood parameters over time across treatment groups. A quadratic term for time was included in the models because of the non-linear trends observed in blood parameters over time. These models also account for variability in baseline values across mice, which were analyzed for trends as well.

## Figures and Tables

**Table 1:** The number of mice and the different treatments that were carried out in the different studies following lethal radiation, with treatment started 24 h after lethal-dose exposure.

Experiment	Treatment	Total Number in the Survival Experiment	Scheduled Necropsy	Euthanized or Found Dead	Survived Through the End of Experiment
#1	sPIF	8	2	2	6
	Scrambled PIF	1	2	0	1
#2	sPIF	13	2	10	3
	Scrambled PIF	3	2	3	0
	Irradiated– no treatment (control)	3	2	0	3
#3	sPIF	38	0	23	15
	Scrambled PIF	15	0	12	3
	PBS	15	0	14	1
Treatment		Total used for calculation	Total Euthanized or Found Dead		Total Survived
sPIF		**59**	35		24
Sham		34	29		5

## Data Availability

The data presented in this study are available upon request from the corresponding author.

## Supplementary Materials

The following supporting information can be downloaded at https://www.mdpi.com/article/10.3390/ijms27177548/s1.

## Abbreviations

The following abbreviations are used in this manuscript:

GI	gastrointestinal
ARS	acute radiation syndrome
FDA	Food and Drug Administration
GvHD	graft-versus-host disease
H-ARS	hematopoietic acute radiation syndrome
HSCT	hematopoietic stem cell transplantation
IR	ionizing radiation
LGFs	leukocyte growth factors
scrPIF	scrambled preimplantation factor
sPIF	synthetic preimplantation factor
TBI	total-body irradiation
PIF	preimplantation factor
sPIF	synthetic preimplantation factor
SCsPIF	subcutaneous synthetic preimplantation factor
