# Supplementary material for: Synthetic Preimplantation Factor (sPIF)* Improves Survival and GI Function in Acute Lethal Radiation Syndrome in a Clinically Relevant Murine Model
Source: Int J Mol Sci. 2026 Aug 23;27(17):7548. doi: 10.3390/ijms27177548 (PMC13566414; doi:10.3390/ijms27177548)
Supplement: Supplementary file 1 [file ijms-27-07548-s001.zip › ijms-4396614-supplementary.pdf]

(**A**)

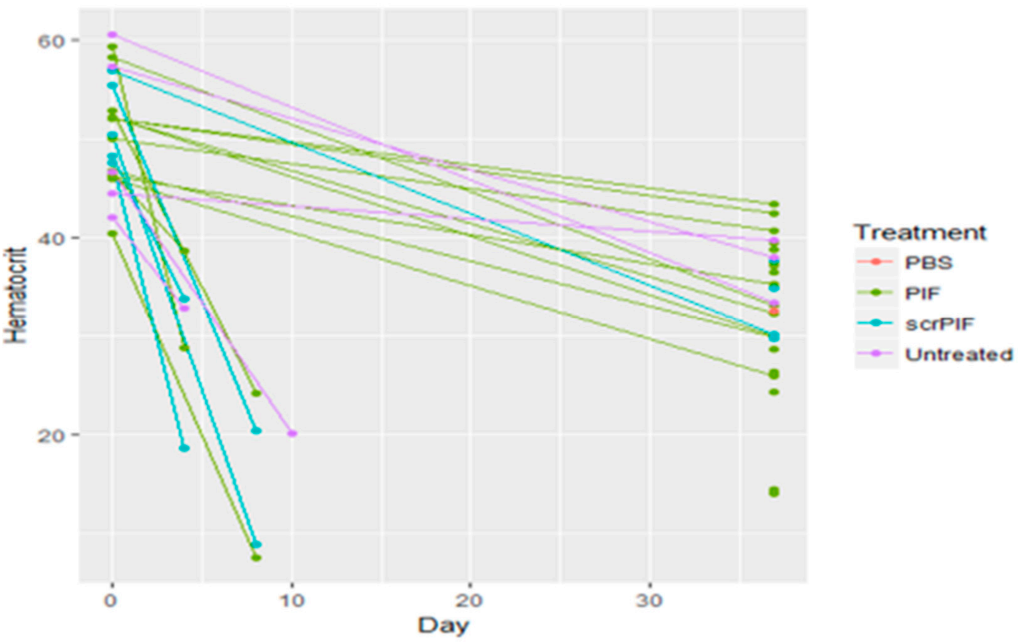
60
40
20
Hematocrit
0
10
20
30
Day
Treatment
PBS
PIF
scrPIF
Untreated

(**B**)

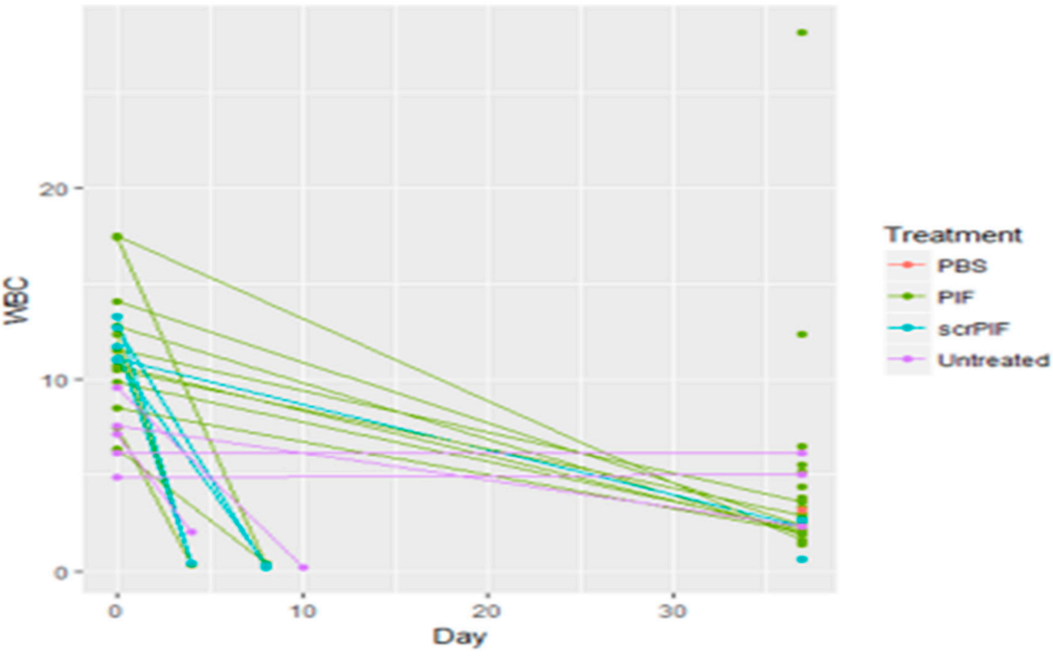
20
10
0
WBC
0
10
20
30
Day
Treatment
PBS
PIF
scrPIF
Untreated

(**C**)

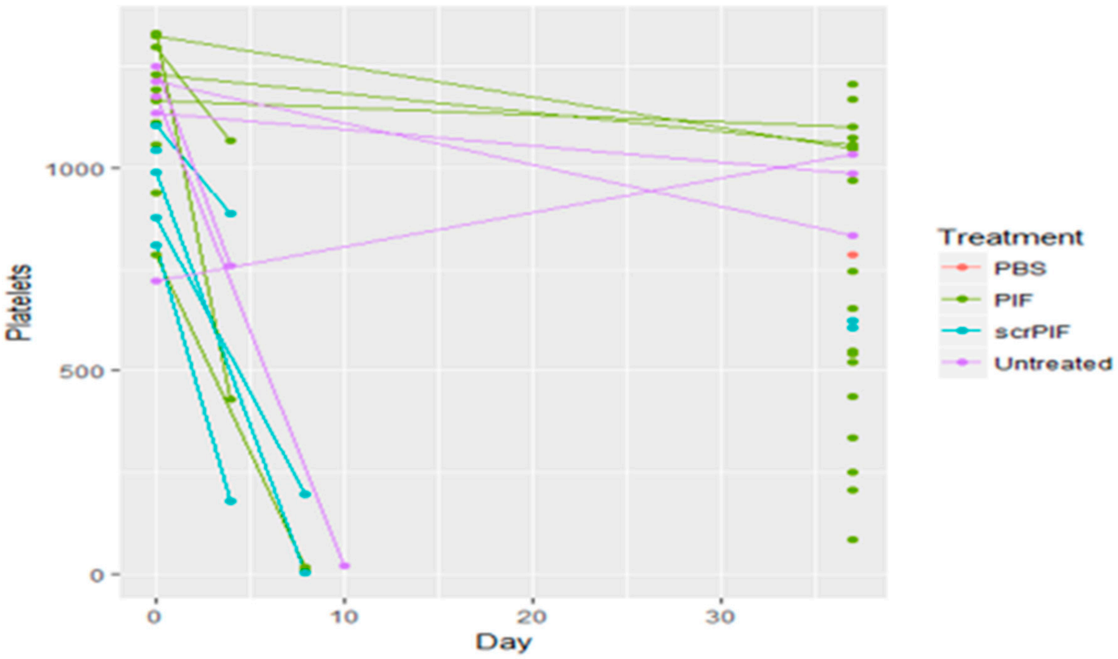

Supplement Figure S1: (**A**) sPIF effects on individual hematocrit (HCT) percent. Shown are HCT levels over time after radiation for mice receiving sPIF, sPIFscr, or PBS. The untreated group (irradiated but not provided with any intervention) was not tested in the final study. Note that, as compared with baseline values, the HCT level decreases only mildly in the sPIF-treated group (13.7+/-6.7% mean+/-SD). (**B**) sPIF effects on the individual platelet count. Shown are platelets that count over time for mice receiving sPIF, sPIFscr, or PBS. The untreated group (irradiated but not provided with any intervention) was not tested in the final study. Compared with baseline values, the platelet count declined by 15-20% in 3 mice for whom both baseline and end-of-study samples were available. (**C**): sPIF effect on the individual total WBC count. Shown are WBC counts for mice receiving sPIF, sPIFscr, or PBS. The untreated group (irradiated but not provided with any intervention) was not tested in the final study. Note that, as compared with baseline values, the WBC count decreases by 65-70% sometime after 8 days. The untreated mice starting WBC count was already <7, and in one case, it dropped to <1.
